# Central ECMO cannulation for severe dihydropyridine calcium channel blocker overdose

**DOI:** 10.1051/ject/2023037

**Published:** 2023-12-15

**Authors:** Jose M. Cardenas, Santiago Borasino, Joseph Timpa, Jeremy Hawkins, Martha McBride, William Rushton, Jordan Newman, Erika Mendoza, Robert Sorabella, Jonathan Byrnes

**Affiliations:** 1 Division of Pediatric Cardiology Section of Cardiac Critical Care. University of Alabama at Birmingham School of Medicine Birmingham AL USA; 2 Department of Cardiovascular Perfusion Children’s of Alabama Birmingham AL USA; 3 ECMO Clinical Coordinator Children’s of Alabama Birmingham AL USA; 4 Department of Pediatric Emergency Medicine. University of Alabama at Birmingham School of Medicine Birmingham AL USA; 5 Department of Pediatric Critical Care. University of Alabama at Birmingham School of Medicine Birmingham AL USA

**Keywords:** ECMO (extracorporeal membrane oxygenation), Shock, Peripheral vascular disease, Pediatric, Pharmacology, cardiovascular

## Abstract

Calcium channel blocker (CCB) toxicity carries a high mortality and is the sixth most fatal drug class reported to US poison centers. Amlodipine overdose is characterized by a life-threatening arterial vasodilation that compromises organ perfusion. The management of CCB intoxication is focused on maintaining adequate organ perfusion. In cases refractory to medical therapies, hemodynamic support with extracorporeal membrane oxygenation (ECMO) is warranted necessitating higher flows than usual to compensate for the vasodilation and requiring central cannulation. We present a case of a 12-year-old with severe dihydropyridine CCB ingestion, refractory to medical management and successfully treated with central ECMO cannulation. The patient was discharged home with no significant disability. Central ECMO cannulation may be helpful to facilitate adequate flows in vasodilatory shock such as CCB overdose.

## Overview

Calcium channel blockers (CCB) are commonly used for the management of hypertension and dysrhythmias. They are generally classified as:

- Non-dihydropyridines such as diltiazem and verapamil preferentially inhibit the L-type calcium channels in the myocardium precipitating negative inotropism and chronotropism. At toxic concentrations, these drugs can induce a vasodilatory shock and bradycardia with poor myocardial contractility.
- Dihydropyridines such as amlodipine, nifedipine, and nicardipine block the L-type calcium channels in peripheral vascular smooth muscle, reducing peripheral vascular resistance. Supratherapeutic ingestion induces a life-threatening vasodilatory shock with reflex tachycardia [1].

However, in massive ingestion, each drug class can lose specificity and precipitate both vasodilatory and cardiogenic shock.

Calcium channel blocker (CCB) toxicity carries a high mortality; recent data published by the American Association of Poison Control Centers reports CCB as the sixth most fatal xenobiotic class making CCBs more lethal than any other antihypertensive agent [2]. Dihydropyridine CCB toxicity management emphasizes re-establishing appropriate organ perfusion with aggressive fluid resuscitation and vasoactive infusions. In addition, drug effects can be ameliorated by administration of intravenous calcium, high-dose insulin and glucose therapy, and lipid emulsion. In cases refractory to medical therapies, hemodynamic support with extracorporeal membrane oxygenation (ECMO) is indicated and due to the distributive shock higher flows than usual may be warranted necessitating central cannulation [3].

Here we report a case of severe dihydropyridine overdose presenting with refractory vasodilatory shock managed with central ECMO cannulation.

## Description

A 12-year-old, 63 kg, previously healthy female, presented to the emergency room for a two-day history of abdominal pain and emesis. Upon evaluation, she was found hypotensive (70/30 mm/Hg), tachycardic (sinus, HR 130 BPM), and hypoxemic (SpO2 85%). Initial ancillary studies were significant for acute kidney injury (serum creatinine 1.6 mg/dL, and BUN 40 mg/dL). Echocardiography confirmed normal anatomy and function, and a chest roentgenogram was significant for basal pulmonary infiltrates. The patient received three liters of isotonic fluid resuscitation, was placed on high-flow oxygen, and was admitted to the pediatric intensive care unit with suspected septic shock.

Following admission, the patient developed anuric renal failure and acute respiratory distress syndrome associated with hemodynamics decompensation (peak lactic acid 10.6 mmol/L). There was no significant improvement following invasive mechanical ventilation, high doses of epinephrine, norepinephrine, and vasopressin infusions (maximum vasoactive-inotropic score 180), broad-spectrum antibiotics, aggressive fluid resuscitation, stress dose steroids, and methylene blue. On Hospital Day (HD) #2, further investigation revealed the patient had intentionally ingested amlodipine 300 mg four days prior. Subsequently, high-dose intravenous insulin, lipid emulsion, and intravenous calcium were started with minimal improvement.

**Figure 1:**
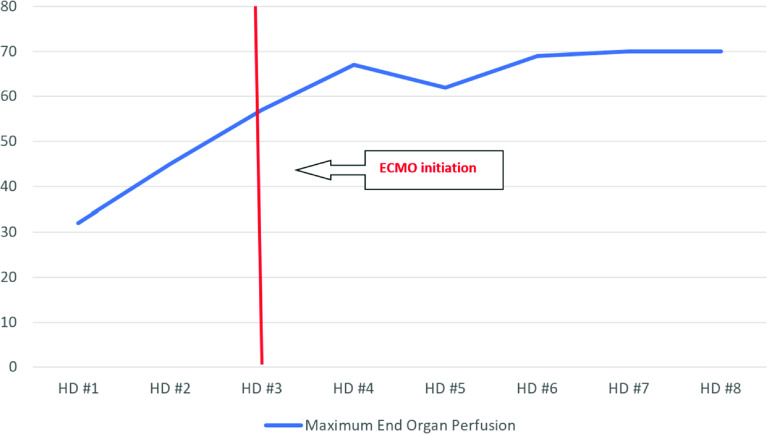
Maximum daily End Organ Perfusion pressure over time. HD: Hospital Day.

**Figure 2:**
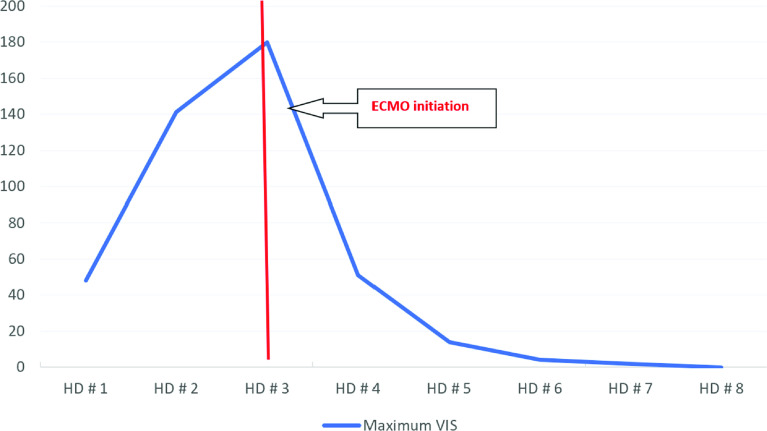
Maximum Vasoactive inotropic score over time. HD: Hospital Day VIS: Vasoactive Inotropic score.

On HD #3, due to worsening multi-organ failure, the patient was centrally cannulated in the operating room for veno-arterial (VA) ECMO due to the high cardiac output state and anticipated limitations in the flow of peripheral ECMO cannulation. A 22-French elongated one-piece arterial aortic cannula (Medtronic) was placed in the ascending aorta and a 36/46-French three-stage cannula (Edwards Lifesciences) was into the right atrial appendage placed by cardiovascular surgery. Shortly after reaching ECMO flows of 7.0 L/min (111 mL/kg/min, Cardiac Index 4 L/min/m^2^), the vasoactive drug requirement was significantly decreased, and urine output improved immediately. Anticoagulation was managed with bivalirudin according to hospital protocol (activated partial thrombin time goal 70-90 seconds). Patient care continued in the pediatric cardiovascular intensive care unit. Figure 1 shows the dramatic drop in vasoactive inotropic score following cannulation and the improvement in organ perfusion pressure is shown in Figure 2.

By HD #6, the vasoactive and mechanical support requirement was minimal. The patient tolerated an ECMO clamp trial well and decannulated with chest closure. She was extubated and weaned off vasoactive on HD#7 and discharged home on HD#21 with no significant disability. Currently, she continues to recover as an outpatient.

## Comment

This case report highlights the utility of central ECMO cannulation in the setting of vasodilatory shock related to dihydropyridine CCB intoxication when conventional medical therapy fails to maintain adequate organ perfusion. To our knowledge, this is the first report of dihydropyridine CCB intoxication managed with central ECMO cannulation prior to circulatory collapse that survived to discharge with a good neurological outcome [3, 4].

Severe amlodipine overdose induces a significant vasodilatory shock due to blockage of L-type calcium channels in the peripheral vasculature with reflex tachycardia- albeit bradycardia and heart block can be present due to loss of receptor selectivity. The management is mainly focused on reestablishing adequate organ perfusion until the drug is metabolized to non-toxic metabolites [3].

The rationale of central cannulation in vasodilatory shock resides in that organ perfusion pressure is driven by the mean arterial pressure (MAP) minus central venous pressure (CVP). MAP is driven by cardiac output (CO) and systemic vascular resistance (SVR). If vasoactive drugs are insufficient to increase the SVR enough to support adequate MAP, further increases in the CO might compensate for low SVR. Therefore, higher CO is required above what conventional ECMO flows provide, and thus larger cannulas through central cannulation may be needed [5]. In our patient, we estimated that a conventional peripheral cannulation would have provided an approximate maximum flow of 5 L/min (80 ml/kg/min, cardiac index 2.9 L/min/m^2^), which would have been insufficient based on the degree of vasodilation and the higher flows required to obtain an adequate organ perfusion (7 L/min). The increased cardiac output provided by high ECMO flows dramatically decreased the vasoactive drug requirement (Figure 1) and significantly improved the end organ perfusion pressure (Figure 2), correlating with a significant improvement in urine output.

Central ECMO cannulation may be helpful to facilitate adequate flows in vasodilatory shock such as dihydropyridine CCB overdose. Limited documentation exists in the use of extracorporeal life support in cases of CCB intoxication, and to our knowledge, this is the first case using central ECMO cannulation with good outcomes for severe intoxication with a dihydropyridine CCB.

## Data Availability

The research data are available on request from the authors.
